# A New Strategy to Investigate the Efficacy Markers Underlying the Medicinal Potentials of *Orthosiphon stamineu*s Benth.

**DOI:** 10.3389/fphar.2021.748684

**Published:** 2021-09-24

**Authors:** Zheng Li, Biao Qu, Lei Zhou, Hongwei Chen, Jue Wang, Wei Zhang, Caifa Chen

**Affiliations:** ^1^ Jiangsu Engineering Research Center of Cardiovascular Drugs Targeting Endothelial Cells, College of Health Sciences, School of Life Sciences, Jiangsu Normal University, Xuzhou, China; ^2^ State Key Laboratory of Natural and Biomimetic Drugs, Peking University, Beijing, China; ^3^ State Key Laboratory of Quality Research in Chinese Medicines, Macau University of Science and Technology, Macau, China; ^4^ School of Public Health (Shenzhen), Sun Yat-sen University, Shenzhen, China

**Keywords:** herbal medicine, efficacy markers, *Orthosiphon stamineus* benth., quality control, phytochemical analysis

## Abstract

*Orthosiphon stamineus* Benth. (OSB) is a well-known herbal medicine exerting various pharmacological effects and medicinal potentials. Owing to its complex of phytochemical constituents, as well as the ambiguous relationship between phytochemical constituents and varied bioactivities, it is a great challenge to explore which constituents make a core contribution to the efficacy of OSB, making it difficult to determine the efficacy makers underlying the varied efficacies of OSB. In our work, a new strategy was exploited and applied for investigating efficacy markers of OSB consisting of phytochemical analysis, *in vivo* absorption analysis, bioactive compound screening, and bioactive compound quantification. Using liquid chromatography coupled with mass spectrometry, a total of 34 phytochemical components were detected in the OSB extract. Subsequently, based on *in vivo* absorption analysis, 14 phytochemical constituents in the form of prototypes were retained as potential bioactive compounds. Ten diseases were selected as the potential indications of OSB based on previous reports, and then the overall interaction between compounds, action targets, action pathways, and diseases was revealed based on bioinformatic analysis. After refining key pathways and targets, the interaction reversing from pathways, targets to constituents was deduced, and the core constituents, including tanshinone IIA, sinensetin, salvianolic acid B, rosmarinic acid, and salvigenin, were screened out as the efficacy markers of OSB. Finally, the contents of these five constituents were quantified in three different batches of OSB extracts. Among them, the content of salvianolic acid B was the highest while the content of tanshinone IIA was the lowest. Our work could provide a promising direction for future research on the quality control and pharmacological mechanism of OSB.

## Introduction

The usage of herbal products as drugs for disease treatment, cosmetics, and health supplements is truly universal as can be seen in different regions of the world. Moreover, herbs, commonly containing various primary and secondary metabolites, have been exploited as a valuable source of leading compounds, and many of the approved drugs have been directly or indirectly derived from them (Bauer et al., 2014). *Orthosiphon stamineus* Benth. (syn: *Orthosiphon aristatus* (Blume) Miq.) (OSB) is a medicinal plant from the Lamiaceae family which is widely distributed in southern China and Southeast Asia. In China, the dried whole plant of OSB is also named “Shen Cha,” which is a popular folk medicine of Dai nationality (Chen et al., 2009). As a traditional medicinal herb, OSB has been widely used for the treatment of kidney stones and other urinary tract diseases on empiricism (Arafat et al., 2008). In past decades, many studies have explored the pharmacological effects and medicinal potentials of OSB. The reported therapeutic effects of OSB included a diuretic effect for treating urinary diseases (Adam et al., 2009), glucose-lowering ability for treating type 2 diabetes mellitus (T2DM) (Lokman et al., 2019), anti-inflammatory activities for managing arthritis (Tabana et al., 2016), hepatoprotective effect for alleviating liver injury (Yam et al., 2007), and neuroprotective ability for improving Alzheimer’s disease (AD) (Retinasamy et al., 2020), etc.

Many constituents have been identified in this herb and the major compounds included phenolic acids, polymethoxylated flavonoids, terpenoids, hexoses, and saponins (Malterud et al., 1989; Tezuka et al., 2000; Nguyen et al., 2004; Hossain et al., 2013; Guo et al., 2019b). In previous studies, the water-soluble constituents, such as protocatechuic acid, caffeic acid, and danshensu, were regarded as the key bioactive constituents, since they were found to possess anti-oxidant and anti-inflammatory properties (Nuengchamnong et al., 2011; Alshawsh et al., 2012). Meanwhile, the alcohol-soluble constituents including sinensetin, eupatorin, and 3′-hydroxy-5,6,7,4′-tetramethoxyflavone (TMF), were also reported to exert various pharmacological activities, thus the efficacy of OSB has also been attributed to them in several studies (Yam et al., 2008; Yam et al., 2009; Chan et al., 2017). However, there are dozens of constituents in OSB, so many of them have been reported to exhibit bioactivities. Furthermore, the multiple constituents could synergistically act on targets to yield a holistic therapeutic effect. Consequently, it is a great challenge to explore which constituents make a core contribution to the efficacy of OSB. Likewise, these issues also make it difficult to elucidate the relationship between the phytochemicals and the holistic efficacy.

In the most recent decade, bioinformatics, represented by network pharmacology, have become efficient tools for revealing the scientific basis and systematic features of herbal medicines. These approaches provide a holistic insight into the relationship between compounds, targets, and signaling pathways behind drug efficacy (Hopkins, 2008; Chen et al., 2016). Interestingly, recent studies have utilized network analysis to screen bioactive constituents as efficacy markers for quality control of herbal medicines (Liao et al., 2018; Xiang et al., 2018; Luo L. et al., 2020). However, challenges are still hanging over many of those related studies. For instance, the predicted phytochemicals from databases could show significant deviation from the realistic constituents of herbs. Moreover, the latest analytical techniques might provide more and newer phytochemicals which go beyond the content of databases. The predicted oral bioavailability (OB) is often used in preliminary screening of phytochemicals, whereas, the screened constituents might differ from the constituents actually absorbed in blood. In addition, one herb could have medicinal potentials for the treatment of different diseases. Nevertheless, often, many studies only focus on one indication, thus resulting in biased conclusions of bioactive constituents to one specific disease. Consequently, these constituents fail to account for the overall efficacy of the herb, which results in the fragile reliability of determining them as efficacy markers.

In this work, a new strategy, consisting of phytochemical analysis, *in vivo* absorption analysis, bioactive compound screening, and bioactive compound quantification, was exploited for investigating efficacy markers underlying the medicinal potentials of OSB. Using liquid chromatography coupled with quadrupole time-of-flight mass spectrometry (LC-Q/TOF-MS), the constituents in OSB extract were comprehensively characterized. Then, the blood-absorbed constituents were identified in plasma samples from rats after oral administration of OSB extract, which were retained as potential bioactive compounds. After selecting the potential indications of OSB, bioinformatic analysis was used to reveal the interaction between compounds, action targets, action pathways, and different diseases, and the core bioactive constituents were screened out as the efficacy markers of OSB. Finally, the quantitative analysis of efficacy markers was carried out by an established LC-MS/MS method.

## Materials and Methods

### Reagents and Materials

Raw materials of OSB were purchased from Yunnan Jianping Biotechnology Co., Ltd. (Origin: Xishuangbanna, Yunnan, China). Analytical grade ethanol and chloroform were obtained from Anaqua Global International Inc. Limited (Cleveland, OH, United States). LC-MS-grade acetonitrile was supplied from J. T. Baker (Phillipsburg, NJ, United States of America). LC-MS-grade formic acid was obtained from Fisher Scientific (Fair Lawn, NJ, United States). Ultra-pure water produced from a Milli-Q Gradient Water System (Millipore Corp Bedford, United States) was used throughout the study. Chromatographic column Sepax GP-C18 (2.1 × 150 mm, 1.8 µm) was purchased from Sepax Technologies (Newark, DE, United States). Reference substances of protocatechuic acid and cichoric acid were obtained from National Institutes for Food and Drug Control (Beijing, China); danshensu, rosmarinic acid, salvianolic acid A, salvianolic acid B, and sinensetin were purchased from Macklin Biochemical Co., Ltd. (Shanghai, China); eupatorin, salvigenin, and TMF were provided by Shanghai yuanye Bio-Technology Co., Ltd. (Shanghai, China). All reference substances possessed high purities up to 97%.

### Preparation of OSB Extract Samples

The dried herb of OSB was smashed to powder. A total of 10 g powder was extracted twice with 100 ml of ethanol-water (80:20, v/v) in an ultrasonic bath for 30 min. The mixture was filtered and the filtrate was combined. The obtained solution was evaporated to a concrete under reduced pressure at 55°C. Then it was dissolved in methanol to remove starch and polysaccharides. After standing at room temperature for 24 h, the solution was centrifuged to remove the precipitate (12,000×*g*, 15 min). The obtained filtrate was evaporated under reduced pressure at 40°C until dry to yield the OSB extract. For qualitative analysis, OSB extract (3.0 mg) was dissolved in 10.0 mL of methanol, followed by filtration through a 0.22 µm nylon membrane filter before LC-MS analysis. Respective standard stock solutions of eight reference substances were prepared in methanol, and stored at −20°C before use.

### Preparation of OSB Plasma Samples

Male Sprague-Dawley rats (200 ± 20 g, *n* = 3) were provided by Guangdong Medical Laboratory Animal Center (Guangzhou, China), and fed at the Experimental Animal Center of Macau University of Science and Technology (Macau, China). Rats were kept at an ambient temperature of 22–25°C and a relative humidity of 55 ± 5% with 12 h light/dark cycles. They were fed with free access to water and food, and fasted with free access to water for 12 h before drug administration. The experimental protocol was approved by the Ethics Review Committee for Animal Experimentation of Macau University of Science and Technology. All procedures were in accordance with the Guide for the Care and Use of Laboratory Animals (National Institutes of Health). OSB extract was dissolved in 0.5% carboxymethyl cellulose sodium aqueous solution to give an apparent concentration of 0.2 g/mL for oral administration. Then the OSB extract was administered to rats orally with a single dose of 1.0 g/kg. Blood samples were then collected from the tail vein at 1 h after administration and centrifuged to separate plasma. A total of 100 μL of plasma was mixed with methanol/acetonitrile (1:2; v:v) solution (300 μL), followed by vortex for 1 min. After centrifugation, the collected supernatant was evaporated until dry under nitrogen gas. The residue was re-dissolved in 200 μL of methanol and then centrifuged to separate the supernatant for LC-MS analysis.

### Qualitative Analysis of Phytochemical Constituents in OSB Samples

Qualitative analysis was carried out on an Agilent 6550 ultra-performance liquid chromatography coupled with a quadrupole time-of-flight mass spectrometry (UPLC-Q/TOF-MS) system. Chromatographic separation was achieved on a Sepax GP-C18 column with an ambient temperature of 35°C. The mobile phase was composed of 0.1% formic acid aqueous solution (A) and acetonitrile (B), and delivered at a flow rate of 0.25 mL/min using the following gradient program: 0–5 min, 25–35% B; 5–10 min, 35–50% B; 10–15 min, 50–53% B; 15–20 min, 53–56% B; 20–25 min, 56–80% B; 25–30 min, 80–85% B; 30–35 min, 85–90% B; 35–40 min, 90–95% B; 40–45 min, 95–25% B. The autosampler was set at 4°C and the injection volume was 5 μL. The MS equipped with an electrospray ionization (ESI) source was carried out in both positive and negative modes using the following optimized parameters: ion spray voltage, 3500 V for positive mode and 3000 V for negative mode; vaporizer temperature, 280°C; sheath gas pressure, 50 psi; capillary temperature, 320°C; and auxiliary gas pressure, 15 psi. The full scan data were acquired from 100 to 1,000 Da, and MS/MS fragmentation was carried out with different collision energy. Identification of the phytochemical compounds was achieved by matching their retention times (RT), molecular ions, and product ions obtained from LC-MS and LC-MS/MS analysis with corresponding reference substances and literature data.

### Constituent Screening and Target Collection

For constituents detected in OSB extract, the OB parameters were extracted from the TCMSP database (http://tcmspw.com/index.php), and OB ≥ 30% was selected as a threshold for screening potential bioactive constituents. For constituents detected in plasma samples, all of them were considered as potential bioactive constituents. Based on comparison, the potential active constituents were determined. Then, the TCMSP (http://tcmspw.com/index.php) and STITCH (http://stitch.embl.de/) databases were used to predict potential action targets of OSB. Moreover, a text mining of PubMed (2016–2021) with each constituent as a search term was carried out to manually extract potential targets for updates and supplementation.

Based on the reported efficacies of OSB, ten diseases were chose as its potential clinical indications, including AD, arthritis, chronic glomerulonephritis, chronic renal failure, gout, hepatic cirrhosis, hepatic fibrosis, hyperlipidemia, nephrolithiasis, and T2DM. The targets of these diseases were extracted from the human gene database GeneCards (http://www.genecards.org/). The items “Symbol” and “Score” of genes were reserved, in which the “Score” represents the relevance degree between disease and target. The intersection targets between OSB and each disease were generated through target mapping. The contribution of each constituent to the intersection targets was analyzed.

### Bioinformatic Analysis of Compounds, Targets, Enriched Pathways, and Their Interaction

Since different constituents in OSB had the same targets, the repetitive targets were merged and the repetition quantity of each target was counted as *n*, followed by normalization with the maximum normalized to 1. For each disease, the “Score” values of their targets were also normalized by being divided by the maximum. Subsequently, the quantitative datasets of OSB targets and disease targets were combined to build a data matrix of OSB-disease targets. Then it was imported into software SIMCA-P (Ver.12.0) and Heml (Ver.1.0.3) for multivariate data analysis to investigate the relevance degree between OSB target profile and disease target profile.

The target data of each OSB-disease pair were combined to generate a whole dataset. After it was imported into Cytoscape (Ver.3.7), the network visualizing the relationship of OSB-compound-target-disease was constructed. For each OSB-disease pair, the top 7 constituents and top 20 targets were extracted according to their degree values. For each OSB-disease pair, the intersection targets were imported into STRING (http://www.string-db.org/) to predict the protein-protein interaction (PPI). Subsequently, the top 20 targets from each PPI network were extracted and combined. These targets were used to generate a new PPI network, and the key regulatory targets were predicted. KEGG pathway enrichment was performed, and a *p* value was given along with each enriched pathway. Based on *p* values, the top 20 pathways were extracted for each OSB-disease pair, respectively. Then these top pathways were combined, and among them the shared pathways by all OSB-disease pairs were retained. Meanwhile, the embedded targets and signaling pathways were also labeled.

### Screening the Efficacy Markers of OSB Through Reversing Bioinformatics Analysis

Based on the enriched pathways, the raw data of key KEGG pathways consisting of embedded signaling pathways and targets were retrieved. After PPI analysis, the raw data of the key target-target interaction were retrieved. From the compound-target network, the raw data of the compound-target interaction were retrieved. Subsequently, the retrieved datasets were combined to generate a new whole dataset. After it was imported into Cytoscape (Ver.3.7), a comprehensive network, that strung together pathway-target pairs, target-target pairs, and target-compound pairs, was finally constructed. Based on topological analysis, the core signaling pathways were firstly deduced then the core targets, and finally the core constituents were screened out as the efficacy markers of OSB.

### Quantitative Analysis of the Efficacy Markers in OSB

Liquid chromatography coupled with triple-quadrupole mass spectrometry (LC-QQQ-MS/MS) was used for simultaneous quantitation of efficacy markers in OSB. A Waters ACQUITY BEH C18 column (3.0 × 100 mm, 2.5 µm) was used for chromatographic separation. The column temperature was maintained at 35°C. Mobile phase consisted of 0.1% formic acid solution (A) and acetonitrile (B). To obtain a short run time and good chromatographic behaviors, the LC conditions were optimized. The flow rate was set at 0.3 mL/min using the following gradient program: 0–5 min, 5–35% B; 5–7 min, 35–60% B; 7–8 min, 65–70% B; 8–9 min, 70–90% B; 9–10 min, 90–5% B. ESI-MS/MS was carried out in both positive and negative modes. The ESI parameters were as follows: ESI temperature was 500°C; ion spray voltage was 5500 V and 4500 V in the negative and positive modes, respectively; curtain gas was 20 psi. Meanwhile, the MS/MS parameters for analyte determination in multiple reaction monitoring (MRM) mode were optimized. The calibration curves were created by running mixed standards of efficacy markers at a series of concentrations. The contents of each efficacy marker in three batches of OSB extracts were determined based on the calibration curve created on the same day.

## Results

### Constituent Identification and Screening in OSB

**FIGURE 1:**
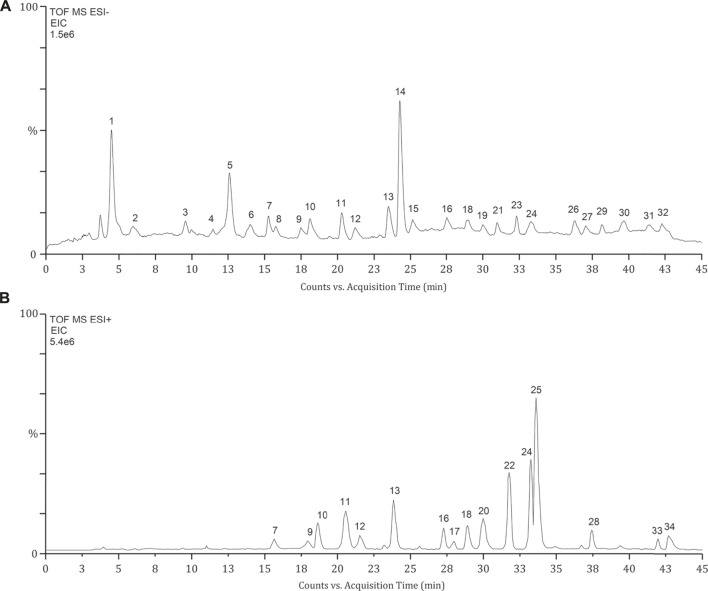
Extracted ion chromatograms (EIC) of OSB samples obtained from LC-Q/TOF-MS analysis. EIC of OSB extract samples in negative mode **(A)** and positive mode **(B)**. LC-Q/TOF-MS, liquid chromatography coupled with quadrupole time-of-flight mass spectrometry; OSB, *Orthosiphon stamineus* Benth.

The identification of phytochemical constituents in OSB extract was performed by LC-Q/TOF-MS analysis. As shown in chromatograms, a larger peak number was generated in the negative mode (Figure 1A) while a stronger MS response was obtained in the positive mode (Figure 1B). The detailed fragment information of parent ions was obtained from MS/MS fragmentation. For these detected peaks, they were deduced and identified based on the retention time, exact mass, and fragment information through matching with reference substances or related data reported in the literature (Malterud et al., 1989; Awale et al., 2001; Akowuah et al., 2004; Guo et al., 2019a; Guo et al., 2019b). As represented in Table 1 and Figure 1, a total of 34 peaks with significant responses were detected. For peaks 1, 3, 8, 10, 12, 14, 18, 22, 24, and 28, these compounds were unambiguously identified by comparison with their reference substances. The identified constituents were categorized as 12 phenylpropenoic acids, 2 benzoic acids, 1 flavonoid glycoside, 12 polymethoxylated flavones, 6 diterpenes, and 1 triterpenoid. Among them, phenylpropenoic acids and polymethoxylated flavones were the top two categories in the OSB extract. Most phenylpropenoic acids displayed the same fragment ion at m/z 179 which was the precursor ion of caffeic acid, and these compounds were regarded as its derivatives. For polymethoxylated flavones, most of them presented same fragment ions at m/z 313 and 298 that were produced by continuous loss of OCH_3_. For diterpenoids, some compounds presenting the same fragment ion at m/z 121 due to the presence of the benzoyl group belonged to the orthosiphol class, and some compounds giving the same fragment ion at m/z 249 belonged to the tanshinone class. In a word, a total of 34 main phytochemical constituents were identified in the OSB extract.

**TABLE 1:** Constituents identified in the extract of *Orthosiphon stamineus* Benth.

Peak No	Precursor ion	Measured mass (m/z)	Calculated mass (m/z)	Error (ppm)	Formula	Fragments (m/z)	Identification	Categories
1	[M–H]^−^	197.0432	197.0449	8.5	C_9_H_10_O_5_	197, 179, 135, 109	Danshensu	Phenylpropenoic acids
2	[M–H]^−^	179.0335	179.0343	4.6	C_9_H_8_O_4_	179, 135	Caffeic acid	Phenylpropenoic acids
3	[M–H]^−^	153.0173	153.0187	8.9	C_7_H_6_O_4_	153, 109	Protocatechuic acid	Benzoic acids
4	[M–H]^−^	167.0349	167.0343	−3.5	C_8_H_8_O_4_	167, 138, 109	Protocatechuic acid methyl ester	Benzoic acids
5	[M–H]^−^	179.0329	179.0343	7.9	C_9_H_8_O_4_	179, 170, 154, 134	Caffeic acid isomer	Phenylpropenoic acids
6	[M–H]^−^	207.0641	207.0656	7.3	C_11_H_12_O_4_	207, 179, 135	Caffeic acid ethyl ester	Phenylpropenoic acids
7	[M–H]^−^	521.1098	521.1083	−2.9	C_27_H_22_O_11_	521, 323, 197, 161	Orthosiphoic acid A	Phenylpropenoic acids
8	[M–H]^−^	473.0738	473.0725	−2.7	C_22_H_18_O_12_	473, 179, 149	Cichoric acid	Phenylpropenoic acids
9	[M–H]^−^	717.1436	717.1454	2.6	C_36_H_30_O_16_	717, 519, 537	Salvianolic acid E	Phenylpropenoic acids
10	[M–H]^−^	359.0751	359.0766	4.1	C_18_H_16_O_8_	359, 197, 179	Rosmarinic acid	Phenylpropenoic acids
11	[M–H]^−^	537.1049	537.1032	−3.2	C_27_H_22_O_12_	537, 493, 356	Lithospermic acid	Phenylpropenoic acids
12	[M–H]^−^	493.1152	493.1134	−3.7	C_26_H_22_O_10_	493, 295, 267	Salvianolic acid A	Phenylpropenoic acids
13	[M–H]^−^	491.0961	491.0977	3.3	C_26_H_20_O_10_	491, 293	Salvianolic acid C	Phenylpropenoic acids
14	[M–H]^−^	717.1473	717.1454	−2.6	C_36_H_30_O_16_	717, 519, 321, 295	Salvianolic acid B	Phenylpropenoic acids
15	[M–H]^−^	463.0857	463.0875	4.0	C_21_H_20_O_12_	463, 300271	Isoquercitrin	Flavonoid glycosides
16	[M–H]^−^	329.0675	329.0660	−4.5	C_17_H_14_O_7_	329, 245, 151	Rhamnazin	Polymethoxylated flavones
17	[M + H]^+^	313.1090	313.1077	−4.1	C_18_H_16_O_5_	313, 298, 283	Trimethylapigenin	Polymethoxylated flavones
18	[M–H]^−^	357.0956	357.0973	4.8	C_19_H_18_O_7_	357, 345, 296, 269	3′-Hydroxy-5,6,7,4′-tetramethoxyflavone	Polymethoxylated flavones
19	[M–H]^−^	313.0724	313.0711	−4.2	C_17_H_14_O_6_	313, 299, 285	Ermanin	Polymethoxylated flavones
20	[M + H]^+^	343.1171	343.1183	3.5	C_19_H_18_O_6_	343, 329, 315	Tetramethoxyluteolin	Polymethoxylated flavones
21	[M–H]^−^	313.0720	313.0711	−2.9	C_17_H_14_O_6_	313, 298, 283	Cirsimaritin	Polymethoxylated flavones
22	[M + H]^+^	373.1270	373.1288	4.9	C_20_H_20_O_7_	373, 358, 343, 315	Sinensetin	Polymethoxylated flavones
23	[M–H]^−^	569.2360	569.2386	4.5	C_31_H_38_O_10_	569, 121	Orthosiphol I	Diterpenoids
24	[M–H]^−^	343.0833	343.0817	−4.8	C_18_H_16_O_7_	343, 328, 313, 285	Eupatorin	Polymethoxylated flavones
25	[M + H]^+^	343.1173	343.1183	2.9	C_19_H_18_O_6_	343, 328, 313, 285	Tetramethylscutellarein	Polymethoxylated flavones
26	[M–H]^−^	313.0723	313.0711	−3.8	C_17_H_14_O_6_	313, 298, 83, 255	Pilloin	Polymethoxylated flavones
27	[M–H]^−^	327.0881	327.0867	−4.1	C_18_H_16_O_6_	327, 313, 298	5-Hydroxy-3′,4′,7-trimethoxyflavone	Polymethoxylated flavones
28	[M + H]^+^	329.1038	329.1026	−3.6	C_18_H_16_O_6_	329, 314, 296	Salvigenin	Polymethoxylated flavones
29	[M–H]^−^	631.2527	631.2542	2.4	C_36_H_40_O_10_	631, 121	Orthosiphol N	Diterpenoids
30	[M–H]^−^	675.2828	675.2804	−3.5	C_38_H_44_O_11_	675, 637, 589, 505	Orthosiphol A	Diterpenoids
31	[M–H]^−^	677.2573	677.2597	3.5	C_37_H_42_O_12_	677, 631, 121	Norstaminol A	Diterpenoids
32	[M–H]^−^	455.3540	455.3524	−3.5	C_30_H_48_O_3_	455	Oleanolic acid	Triterpenoids
33	[M + H]^+^	277.0877	277.0866	−4.0	C_18_H_12_O_3_	277, 249, 231	Tanshinone A	Diterpenoids
34	[M + H]^+^	295.1346	295.1335	−3.6	C_19_H_18_O_3_	295, 277, 249	Tanshinone IIA	Diterpenoids

**FIGURE 2:**
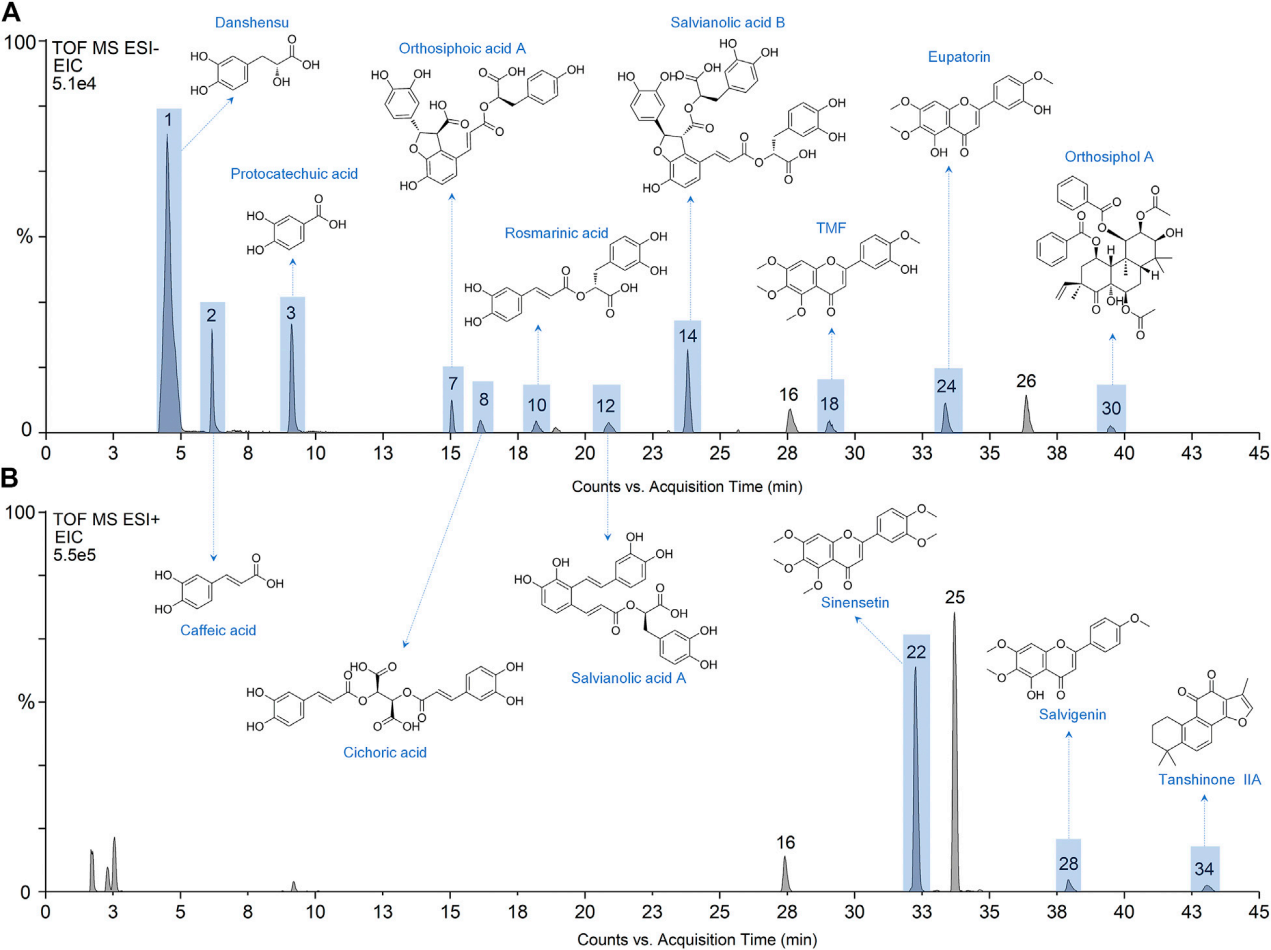
Extracted ion chromatograms (EIC) of plasma samples from rats after administration of OSB extract in negative mode **(A)** and positive mode **(B)**. OSB *Orthosiphon stamineus* Benth.

The developed method was then applied to investigate the blood-absorbed phytochemicals in rats after oral administration of OSB extract. Figures 2A,B showed the representative chromatograms of plasma samples generated in negative and positive modes, respectively. A total of 17 phytochemical constituents of OSB were identified in rat plasma samples. These constituents including danshensu, caffeic acid, protocatechuic acid, orthosiphoic acid A, cichoric acid, rosmarinic acid, salvianolic acid A, salvianolic acid B, rhamnazin, TMF, sinensetin, eupatorin, tetramethylscutellarein, pillion, salvigenin, orthosiphol A, and tanshinone IIA. Consistent with the above phytcochemical characterization, phenylpropenoic acids and polymethoxylated flavones were also the top two categories that could be absorbed in the circulatory system.

Based on the OB parameter, a total of 20 constituents were screened out from 34 constituents. Several constituents with large OB, such as trimethylapigenin (OB 39.83%), however, were not detected in rat plasma. By contrast, several constituents with small OB, such as rosmarinic acid (OB 1.38%), however, were detected in rat plasma. Nevertheless, these screened constituents still showed a lot of overlapping with the phytochemicals detected in plasma samples. For the constituents detected in plasma samples, preliminary collection of their targets was carried out. Finally, the constituents with reported targets were screened out as potential bioactive constituents, including protocatechuic acid, danshensu, caffeic acid, orthosiphoic acid A, cichoric acid, rosmarinic acid, salvianolic acid A, salvianolic acid B, TMF, sinensetin, eupatorin, salvigenin, orthosiphol A, and tanshinone IIA. The chemical structures of these phytochemicals were provided in Figures 2A,B.

### Bioinformatic Analysis of the Interaction From Compounds to Targets and Pathways

**FIGURE 3:**
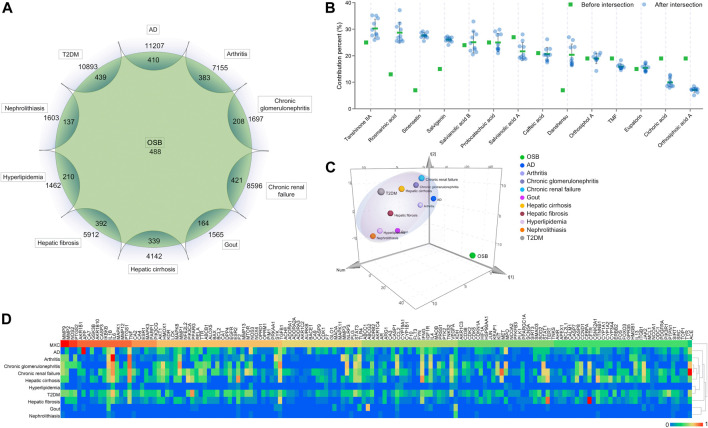
Analysis of the intersection targets between OSB and different diseases. **(A)** Sketch profile of intersection targets between OSB and different diseases; **(B)** Contribution of each constituent to the intersection targets of all OSB-disease pairs; **(C)** 3D score plot generated in SIMCA software using the PCA model for visualizing the overall relevance of targets between groups; **(D)** Heatmap integrating the HCA model for visualizing the relevance of both individual and overall targets between groups. AD, Alzheimer’s disease; HCA, hierarchical cluster analysis; OSB, *Orthosiphon stamineus* Benth.; PCA, principal component analysis; T2DM, type 2 diabetes mellitus.

OSB constituents generated a total of 488 potential targets. After overlapping, OSB shared 410, 383, 208, 421, 164, 339, 392, 210, 137, and 439 targets with AD, arthritis, chronic glomerulonephritis, chronic renal failure, gout, hepatic cirrhosis, hepatic fibrosis, hyperlipidemia, nephrolithiasis, and T2DM, respectively (Figure 3A). As detailed in each phytochemical constituent, the contribution to targets varied from constituent to constituent. Salvianolic acid A, protocatechuic acid, and tanshinone IIA were the top three in terms of counting their contribution to the total targets of OSB (Figure 3B). By comparison, there was a remarkable variation in the overall profile of target contribution after intersection with disease targets (Figure 3B). Rosmarinic acid, sinensetin, salvigenin danshensu, cichoric acid, and orthosiphoic acid A were the representatives, in which the first four constituents showed increased contribution after intersection while the last two showed decreased contribution after intersection. Tanshinone IIA, rosmarinic acid, and sinensetin were the top three constituents in contribution percentage after intersection, in contrast, rosmarinic acid and sinensetin showed a very low contribution percentage before intersection. These results suggested that, for constituents, the effective targets they possessed for diseases were more meaningful than the amount of total targets.

Based on multivariate data analysis employing principal component analysis (PCA) and hierarchical cluster analysis (HCA) models, the relevance degree between OSB target profile and disease target profile was investigated. A 3D score plot was generated in SIMCA using the PCA model, and it visualized the relevance of overall targets between groups (Figure 3C). The OSB group showed clear separation from the disease groups, and most disease groups were located in the same dimension. Interestingly, only the AD group was located in the same dimension as the OSB group, and was the nearest to the OSB group, followed by the arthritis and chronic glomerulonephritis groups (Figure 3C). Consistently, the heatmap generated in Heml using the HCA model showed similar results, which visualized the relevance of both individual and overall targets between groups (Figure 3D). Based on integrative comparison, AD, arthritis, and chronic glomerulonephritis had a strong affinity with OSB in terms of target profile.

**FIGURE 4:**
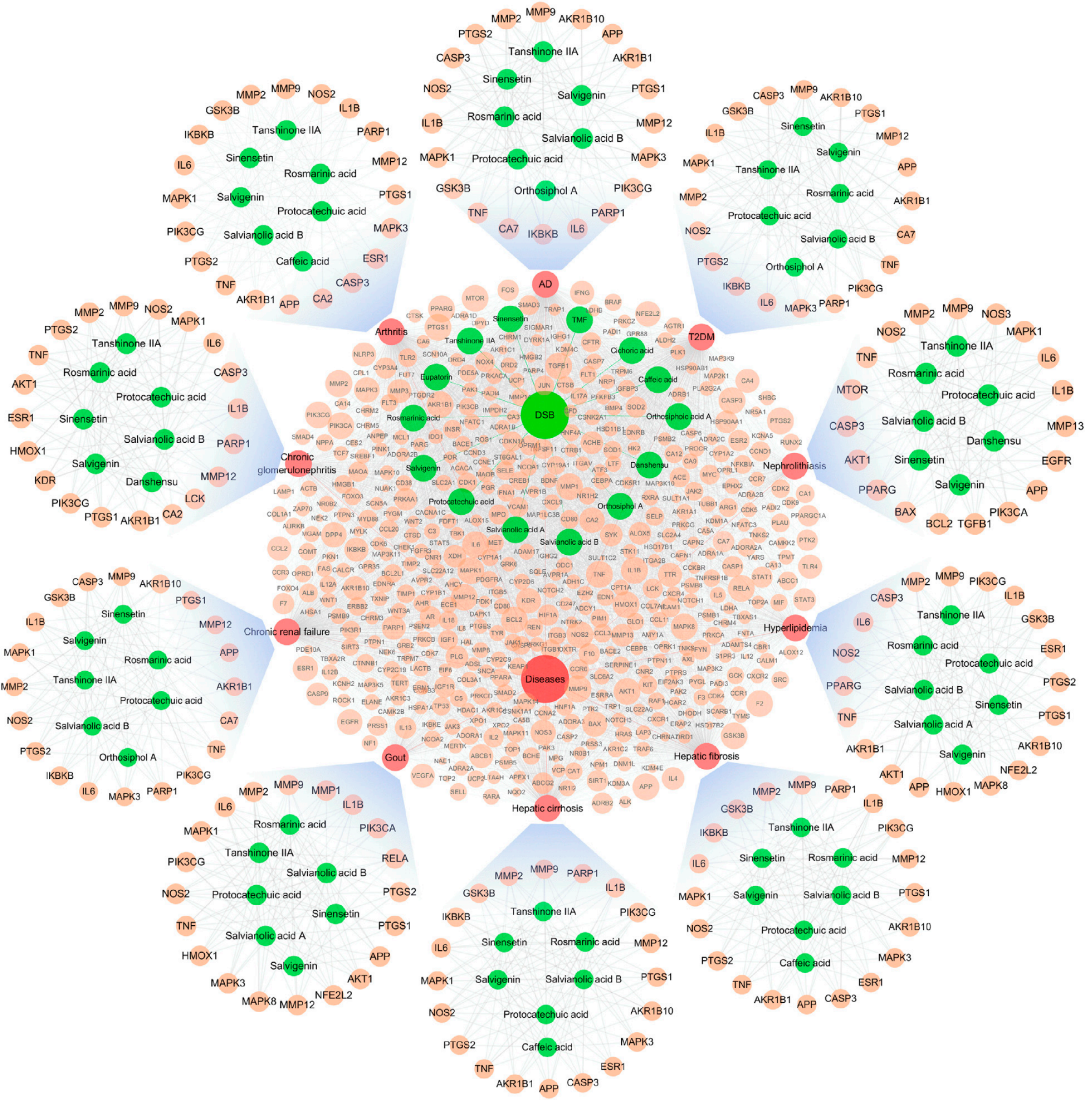
Network visualizing the overall interaction of constituents and targets, with integrated sub-network visualizing the top constituents and targets for each OSB-disease pair. AD, Alzheimer’s disease; OSB, *Orthosiphon stamineus* Benth.; T2DM, type 2 diabetes mellitus. TMF, 3′-Hydroxy-5, 6, 7, 4′-tetramethoxyflavone.

**FIGURE 5:**
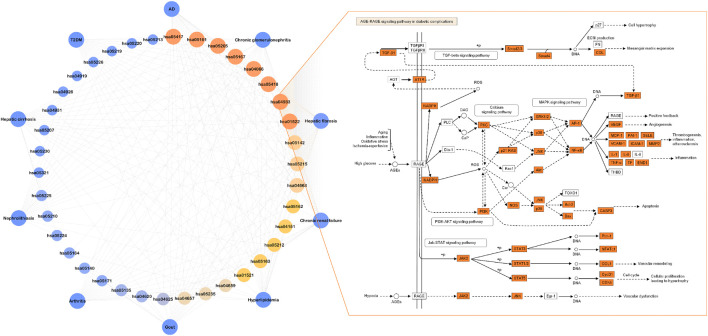
The terms of 37 non-repetitive KEGG pathways retained from the pool of top KEGG pathways for each OSB-disease pair. Among them, eight KEGG pathways were shared by all OSB-disease pairs and a representative KEGG pathway was shown in which OSB-disease intersection targets were labeled with color. hsa05417: lipid and atherosclerosis; hsa05161: hepatitis B; hsa05205: proteoglycans in cancer; hsa05167: kaposi sarcoma-associated herpesvirus infection; hsa04066: HIF-1 signaling pathway, hsa05418: fluid shear stress and atherosclerosis; hsa04933: AGE-RAGE signaling pathway in diabetic complications, hsa01522: endocrine resistance. OSB *Orthosiphon stamineus* Benth.

The interaction of OSB-compound-target-disease was visualized in a network which integrated all OSB-disease pairs (Figure 4). In this network, the top 7 constituents and top 20 targets were magnified into a sub-network for each OSB-disease pair. Among these top constituents, up to six constituents were shared by all OSB-disease pairs as follows: protocatechuic acid, rosmarinic acid, salvianolic acid B, salvigenin, sinensetin, and tanshinone IIA. Among these top targets, up to six targets were shared by all OSB-disease pairs as follows: IL1B, IL6, MMP2, MMP9, NOS2, and TNF. For each OSB-disease pair, the top 20 targets were respectively extracted from the PPI network. Then a refined PPI network was then constructed through integrating these top targets (Supplementary Figure S1). Based on the topological properties, the key regulatory targets were screened out as follows: AKT1, JAK2, JUN, MAPK1, MAPK8, PIK3CA, PIK3R1, RELA, STAT3, TNF, and TP53. After pathway enrichment, the top 20 pathways for each OSB-disease pair were combined. After merging duplicates, only 37 non-repetitive pathways were retained, suggesting extensive overlapping among the top pathways from each OSB-disease pair (Figure 5). After analysis, a total of eight KEGG pathways shared by all OSB-disease pairs were screened out. The detailed information of these pathways is provided in Supplementary Table S1. One representative pathway is shown in Figure 5 in which OSB-disease intersection targets were labeled with color.

### Screening of the Efficacy Markers of OSB Through Reversing Bioinformatics Analysis

**FIGURE 6:**
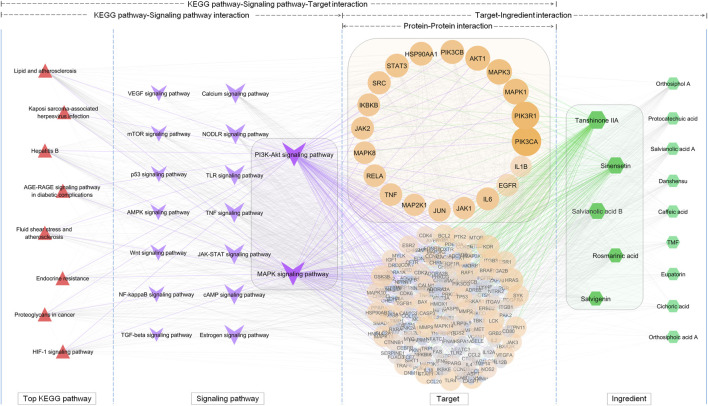
Screening of the efficacy markers underlying the varied medicinal potentials of OSB through reversing bioinformatics analysis which deduced the interaction from key KEGG pathways to signaling pathways, followed by targets, and finally compounds. OSB *Orthosiphon stamineus* Benth.; TMF, 3′-Hydroxy-5, 6, 7, 4′-tetramethoxyflavone.

Based on extraction, 8 key KEGG pathways generated 17 different signaling pathways. As shown in Figure 6, a KEGG pathway contained several signaling pathways, while a signaling pathway was also embedded in several KEGG pathways. These signaling pathways contained 209 OSB-disease intersection targets after merging duplicates. As expected, there was a great overlap of these targets among different signaling pathways. Pathway-target connection was firstly established, and then target-target connection. Based on topological properties, two signaling pathways: PI3K-AKT signaling pathway and MAPK signaling pathway, were screened out as the core action pathways. After integrating target-compound interaction, a full-scale network was constructed which reversely deduced the interaction from KEGG pathways to signaling pathways, then targets, and finally compounds. The topological profile of targets was significantly different from that in the original network as shown in Figure 4. Meanwhile, the core targets were screened out as follows: PIK3CA, PIK3R1, MAPK1, MAPK3, AKT1, PIK3CB, HSP90AA1, IKBKB, MAPK8, and RELA, which also showed a significant difference from the results in Figure 3. Based on topological analysis, the core compounds were finally screened out as follows: tanshinone IIA, sinensetin, salvianolic acid B, rosmarinic acid, and salvigenin. These constituents showed the interaction with all of core targets, and were finally selected as the efficacy markers underlying the various medicinal potentials of OSB.

### Quantitative Analysis of the Efficacy Markers in OSB

The optimized MS/MS parameters in MRM mode for the determination of five efficacy markers were provided in Table 2, including ion transitions, declustering potential (DP), and collision energy (CE). Calibration curve for each analyte was created with a series of concentrations of standard solution. Acceptable linear correlation was confirmed by the correlation coefficient (r, 0.9990–0.9999). The detailed information regarding calibration curves and linear ranges are shown in Table 3. The proposed method was applied in the quantification of five efficacy markers in three different batches of OSB extracts. All compounds were quantified with the content of more than 2.50 mg/g (Table 3). Among them, the content of salvianolic acid B was the highest with the average content of 220.86 mg/g while the content of tanshinone IIA was the lowest with 2.78 mg/g.

**TABLE 2:** MS/MS parameters for analyte determination in MRM mode.

Analytes	Precursor-product ion pairs (m/z)	DP (V)	CE (V)	ESI mode
Tanshinone IIA	295.3 → 277.3	70	28	Positive
Sinensetin	373.1 → 343.0	130	38	Positive
Salvigenin	329.1 → 296.1	110	36	Positive
Rosmarinic acid	359.1 → 161.1	69	20	Negative
Salvianolic acid B	717.1 → 519.1	116	24	Negative

**TABLE 3:** Calibration curves, linear ranges and determined contents of five efficacy markers in OSB extracts.

Compounds	Calibration curves	Linear range (ng/mL)	Contents (mg/g)
Tanshinone IIA	y = 0.001x + 0.0861 (r = 0.9995)	20–1,000	2.78 ± 0.25
Sinensetin	y = 0.0137x − 0.048 (r = 0.9999)	20–1,000	7.18 ± 0.63
Salvianolic acid B	y = 0.0000498x + 0.0109 (r = 0.9996)	200–10,000	220.86 ± 20.92
Rosmarinic acid	y = 0.000551x + 0.0871(r = 0.9990)	100–10,000	129.98 ± 16.49
Salvigenin	y = 0.00373x − 0.0014 (r = 0.9997)	20–1,000	3.01 ± 0.34

## Discussion

As a traditional herbal medicine, OSB has been empirically used for the treatment of urinary tract diseases. Recent studies demonstrated its various medicinal potentials owing to different pharmacological effects, such as anti-oxidation (Nuengchamnong et al., 2011), anti-inflammation (Yam et al., 2010), diuretic (Adam et al., 2009), anti-diabetic (Mohamed et al., 2013), and hepatoprotection (Yam et al., 2007). The phytochemicals in OSB represent the material basis of its pharmacological effects. Taking advantage of modern analytical techniques, it was reported that OSB contained dozens of phytochemical constituents (Malterud et al., 1989; Tezuka et al., 2000; Nguyen et al., 2004; Hossain et al., 2013; Guo et al., 2019b). The constituents in OSB extract could vary with the polarities of extract solvents, which thus has a considerable effect on the bioactivities. Several studies suggested that the aqueous extract fraction of OSB could show weaker bioactivities than the extract fraction with strong polarity (Abdelwahab et al., 2011; Choo et al., 2018). Therefore, large percentage ethanol solution was used as the extract solvent in this study. As a result, a total of 34 constituents were detected in OSB extract. Among them, phenylpropenoic acids, polymethoxylated flavones, and terpenoids represented the predominant structural classes.

The identification of blood-absorbed phytochemicals could be regarded as a preliminary screening of bioactive constituents from oral herbs. Several studies have reported the quantitative analysis of several specified constituents of OSB in rat plasma (Loon et al., 2005; Guo et al., 2019a). However, there is a lack of characterization of the phytochemicals absorbed in the circulatory system. In the present work, a total of 17 phytochemicals were identified in plasma samples from rats after oral administration of OSB extract. On the other hand, the predicted OB parameter, representing the fraction of the orally-administered drug that reaches systemic circulation unchanged, is widely used for preliminary screening of active constituents (Xu et al., 2012). Based on OB, a total of 20 constituents were screened out from 34 constituents that were detected in OSB extract. Interestingly, despite extensive overlapping, a remarkable difference was still found between the two pools of constituents respectively generated by plasma detection and OB screening. Some constituents detected in plasma had small OB, while some constituents with large OB were not actually detected in plasma. These findings suggested that virtual screening using OB resulted in constituent distortion compared with practical detection. In worse cases, this distortion might lead to a cascade of deviation in deduced bioactive constituents and mechanism. Therefore, practical detection of realistic samples could be a more reliable approach.

In following bioinformatic analysis, the key constituents were screened out for each OSB-disease pair based on the degree from topological analysis. This is a well-accepted paradigm for identifying key bioactive constituents in many related studies (Liao et al., 2018; Wang et al., 2019a; Wang et al., 2019b). However, the intersection targets between OSB and diseases varied with paired diseases, thus the screened key constituents could also vary. Furthermore, different targets had varied relevance to diseases as indicated by different “Score” values. However, the targets with weak relevance were treated equally with the targets with strong relevance due to the unbiased strategy. Consequently, a mass of targets with weak relevance, however, might play a prominent part in the screening of key constituents. In our work, the active compounds were screened through reversing bioinformatics analysis. It began with disassembling the enriched key pathways to extract the embedded key targets, thus excluding many targets with weak relevance. After re-constructing the interaction between pathways, targets, and compounds, a new network was generated which was significantly different from the original target-compound network. The core targets in the new network were mainly involved in the PI3K-AKT signaling pathway and MAPK signaling pathway. There was considerable overlapping between the two signaling pathways regarding the contained targets, and the NF-κB signaling pathway was one of the important intersections between them. Now there is no clear clue to the relationship between deduced action mechanisms (PI3K-AKT signaling pathway/MAPK signaling pathways) and traditional therapeutic effects of OSB owing to the limited modern pharmacological studies regarding OSB. However, the NF-κB signaling pathway was reported as the action pathway of OSB accounting for its traditional pharmacological activities in several studies (Li et al., 2016; Retinasamy et al., 2020). Undoubtedly, more effort should be put into the investigation of the precise therapeutic mechanism of OSB. Moreover, the core targets showed minor overlapping with that in the original network. These results suggested that the unbiased strategy for all targets might lead to significant distortion of screened core targets. The core constituents in the new network showed large overlapping with that in the original network, although a slight deviation was still found especially in terms of compound sorting. These results demonstrated the robustness of the core bioactive constituents of OSB for the treatment of different diseases, which were extremely appropriate as the efficacy markers of OSB.

Bioactive constituents including tanshinone IIA, sinensetin, salvianolic acid B, rosmarinic acid, and salvigenin, were selected as the efficacy markers accounting for the varied efficacies of OSB. Surprisingly, in previous OSB-related studies, there were few points of focus on tanshinone IIA. The major interest was fastened on rosmarinic acid and flavonoids (Yuliana et al., 2009; Shafaei et al., 2016; Cai et al., 2018). Only recently, one study that aimed at qualitative and quantitative analysis of phytochemicals in OSB characterized tanshinone IIA in OSB extract (Guo et al., 2019b). In contrast to its nonentity in previous OSB-related studies, tanshinone IIA has been reported to exert various therapeutic activities, such as improving renal function (Zhang et al., 2020), relieving myocardial ischemia reperfusion injury (Li Q. et al., 2016), alleviating neuroinflammation (Maione et al., 2018), and exerting an antifibrotic effect (Shi et al., 2020). The action mechanism was suggested to be mainly involved in activating the PI3K-AKT signaling pathway and/or suppressing the MAPK and NF-κB signaling pathways. This evidence underlined the reliability for selecting tanshinone IIA as an efficacy marker of OSB. In previous studies, the constituents selected as efficacy contributors of OSB, varied with studied diseases. Even caffeic acid and protocatechuic acid, which widely exist in a variety of herbs and lack specificity, were also regarded as the core bioactive constituents and included in the quality control of OSB (Guo et al., 2019a; Guo et al., 2019b). Nevertheless, in most studies, rosmarinic acid, salvianolic acid B, and methoxy flavonoids were widely accepted as the bioactive constituents of OSB (Loon et al., 2005; Nuengchamnong et al., 2011; Cai et al., 2018; Yam et al., 2018), which was consistent with our results. Indeed, these phytochemical monomers have been reported with definite pharmacological effects for the treatment of various diseases (Mohamed et al., 2012; Tongqiang et al., 2016; Serino et al., 2021; Yamamoto et al., 2021). Rosmarinic acid is contained mainly in the family Lamiaceae, and numerous studies have demonstrated its health benefits, especially in management of inflammatory diseases via inhibition of oxidative stress, apoptosis, and inflammation (Joardar et al., 2019; Luo et al., 2020). Salvianolic acid B was remarkably abundant in OSB, and several studies indicated that it had strong anti-inflammatory and anti-fibrotic effects through targeting the MAPK and NF-κB pathways (Li et al., 2019; Wu et al., 2019). Polymethoxylated flavonoids, such as sinensetin, have been widely reported with anti-inflammatory, anti-oxidant, anti-dementia, and vasorelaxant activities and the action mechanisms could be involved in the regulation of different targets and signaling pathways including AKT and NF-κB signaling pathways (Lee et al., 2020). In sum, these reported evidence strengthened the reliability of selecting these phytochemicals as efficacy makers of OSB.

## Conclusion

In this work, a new strategy was exploited to investigate the efficacy markers underlying the varied pharmacological effects of OSB. By LC-MS analysis, a total of 34 phytochemical constituents were characterized in OSB, and 14 blood-absorbed phytochemicals were retained as potential active compounds. The results from bioinformatic analysis revealed the overall interaction between compounds, action targets, action pathways, and diseases. Through refining key pathways and targets, the interaction reversing from signaling pathways, targets to constituents was deduced, and then the core signaling pathways, targets, and compounds were screened out. Five constituents, including tanshinone IIA, sinensetin, salvianolic acid B, rosmarinic acid, and salvigenin, were finally selected as the efficacy markers accounting for the efficacies of OSB against different diseases. The corresponding action mechanism was suggested to closely relate with the PI3K-AKT signaling pathway and/or MAPK signaling pathway, but further experimental studies are necessary to validate the deduced mechanism. Finally, the contents of the five efficacy markers in OSB extracts were quantified, and the content of salvianolic acid B was the highest while the content of tanshinone IIA was the lowest. It is believed that our findings could provide promising directions for future research on the quality control and pharmacological mechanism of OSB.

## Data Availability

The original contributions presented in the study are included in the article/Supplementary Material, further inquiries can be directed to the corresponding authors.
