# Supplementary material for: A New Strategy to Investigate the Efficacy Markers Underlying the Medicinal Potentials of Orthosiphon stamineus Benth
Source: Front Pharmacol. 2021 Sep 24;12:748684. doi: 10.3389/fphar.2021.748684 (PMC8497827; doi:10.3389/fphar.2021.748684)
Supplement: Supplementary file 1 [file DataSheet1.docx]

Supplementary Material


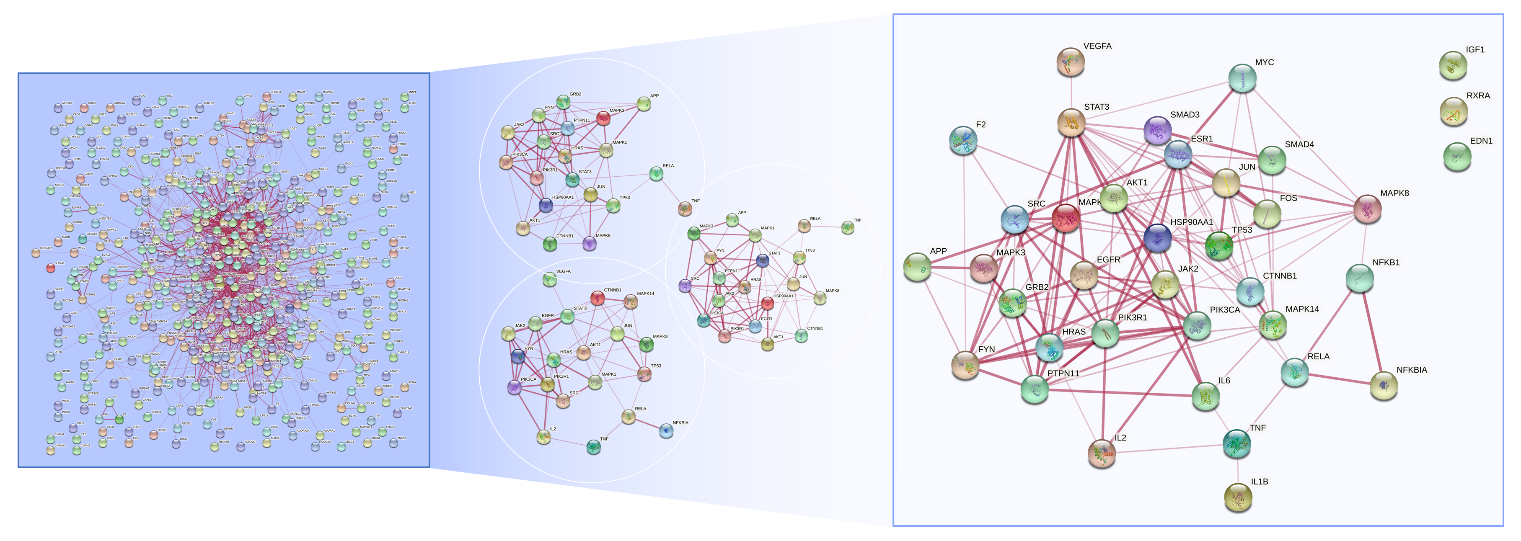


**Supplementary Figure 1** Construction of a refined PPI network through integrating the top targets extracted from the individual PPI network of OSB-disease intersection targets. PPI, protein-protein interaction; OSB, *Orthosiphon stamineus* Benth.

**Supplementary Table 1** Detail information of top KEGG pathways shared by all OSB-disease pairs.

| ID | Description | Gene Ratio | p value | Gene ID |
| --- | --- | --- | --- | --- |
| hsa05417 | Lipid and atherosclerosis | 73/378 | 8.4E-45 | AKT1/BAX/BCL2/BCL2L1/CALM1/CAMK2B/CASP1/CASP3/  CASP6/CASP7/CASP8/CASP9/CCL2/CCL3/CYP1A1/CYP2C9/  EIF2AK3/ERN1/FAS/FOS/GSK3B/HRAS/HSP90AA1/ICAM1/  HSP90AB1/ HSPA1A/IFNA1/IKBKB/IKBKE/IL12A/IL12B/IL18/  IL1B/IL6/JAK2/JUN/MAP3K5/MAPK1/MAPK10/MAPK11/TNF/  MAPK14/MAPK3/MAPK8/MMP1/MMP3/MMP9/MYD88/PTK2/  NFATC1/ NFATC3/NFE2L2/NFKBIA/NLRP3/NOS3/PIK3CA/  PIK3CB/PIK3R1/PPARG/PRKCA/ RELA/RXRA/SELE/ SELP/  SOD2/SRC/STAT3/TBK1/TLR2/TLR4/ TP53/TRAF6/VCAM1 |
| hsa05161 | Hepatitis B | 57/405 | 3.7E-34 | AKT1/BAX/BCL2/BRAF/CASP3/CASP8/CASP9/CCNA2/CDK2/  CCNE1/ CDKN1A/CREB1/FAS/FOS/GRB2/HRAS/IFNA1/IL6/  KBKB/IKBKEJAK1/JAK2/JAK3/JUN/MAP2K1/ IMAPK1/ MYC/  MAPK10/ MAPK11/MAPK14/MAPK3/MAPK8/MMP9/ MYD88/  NFATC1/NFATC3/NFKBIA/PIK3CA/PIK3CB/PIK3R1/ PRKCA/  PRKCB/PRKCG/RAF1/RELA/SMAD3/SMAD4/SRC/STAT1/  STAT3/TBK1/TGFB1/TLR2/TLR4/TNF/TP53/TRAF6 |
| hsa05205 | Proteoglycans in cancer | 59/405 | 6.9E-30 | ACTB/AKT1/BRAF/CAMK2B/CASP3/CCND1/CDKN1A/EGFR/  COL1A1/ ERBB2/ CTNNB1/ESR1/FAS/GRB2/HIF1A/HRAS/  IGF1/IGF1R/IL12B/ITGAV/ITGB1/ITGB3/ITGB5/MAP2K1/KDR/  MAPK1/MAPK11/MAPK14/MAPK3/MET/MMP2/MMP9/MTOR/  MYC/PAK1/PIK3CA/PIK3CB/PIK3R1/PLAU/PRKACA/PRKCA/  PRKCB/PRKCG/PTK2/PTPN11/RAF1/ROCK1/SMAD2/STAT3/  SRC/TGFB1/TLR2/TLR4/TNF/TP53/VEGFA/WNT1/WNT2/WNT3A |
| hsa05167 | Kaposi sarcoma-associated herpesvirus infection | 56/378 | 4.7E-30 | AKT1/BAX/C3/CALM1/CASP3/CASP8/CASP9/CCND1/CCR1/  CCR3/CDK4/CDK6/CDKN1A/CREB1/CTNNB1/FAS/FOS/GSK3B/  HIF1A/HRAS/ICAM1/IFNA1/IKBKB/IKBKE/IL6/JAK1/JAK2/  JUN/MAP1LC3B/MAP2K1/MAPK1/MAPK10/MAPK11/MAPK14/  MAPK3/MAPK8/MTOR/MYC/NFATC1/NFATC3/NFKBIA/PIK3CA/  PIK3CB/PIK3CG/PIK3R1/PTGS2/RAF1/RELA/SRC/STAT1/STAT3/  SYK/TBK1/TCF7/TP53/VEGFA |
| hsa04066 | HIF-1 signaling pathway | 40/405 | 6.0E-25 | AKT1/BCL2/CAMK2B/CDKN1A/EDN1/EGFR/ERBB2/FLT1/  HIF1A/HK2/HMOX1/IFNG/IGF1/IGF1R/IL6/INSR/LDHA/LDHB/  MAP2K1/MAPK1/MAPK3/MTOR/NOS2/NOS3/NPPA/PDK1/PFKFB3/  PIK3CA/PIK3CB/PIK3R1/PRKCA/PRKCB/PRKCG/RELA/  SERPINE1/SLC2A1/STAT3/TIMP1/TLR4/VEGFA |
| hsa05418 | Fluid shear stress and atherosclerosis | 44/378 | 1.5E-25 | ACTB/AKT1/BCL2/BMP4/CALM1/CCL2/CTNNB1/EDN1/FOS/  HMOX1/HSP90AA1/HSP90AB1/ICAM1/IFNG/IKBKB/IL1B/  ITGA2B/ITGAV/ITGB3/JUN/KDR/KEAP1/MAP3K5/MAPK10/  MAPK11/MAPK14/MAPK8/MMP2/MMP9/NFE2L2/NOS3/PIK3CA/  PIK3CB/PIK3R1/PRKAA1/PRKCZ/PTK2/RELA/SELE/SRC/ TNF/  TP53/VCAM1/VEGFA |
| hsa04933 | AGE-RAGE signaling pathway in diabetic complications | 48/405 | 2.0E-36 | AGTR1/AKT1/BAX/BCL2/CASP3/CCL2/CCND1/CDK4/ COL1A1/  COL3A1/EDN1/F3/HRAS/ICAM1/IL1B/IL6/JAK2/JUN/MAPK1/  MAPK10/MAPK11/MAPK14/MAPK3/MAPK8/MMP2/NFATC1/  NOS3/NOX4/PIK3CA/PIK3CB/PIK3R1/PIM1/PRKCA/PRKCB/  PRKCD/PRKCZ/RELA/SELE/SERPINE1/SMAD2/SMAD3/ SMAD4/  STAT1/STAT3/TGFB1/TNF/VCAM1/VEGFA |
| hsa01522 | Endocrine resistance | 40/405 | 4.7E-27 | ADCY1/AKT1/BAX/BCL2/BRAF/CCND1/CDK4/CDKN1A/ CYP2D6/  EGFR/ERBB2/ESR1/ESR2/FOS/GRB2/HRAS/IGF1/IGF1R/JUN/  MAP2K1/MAPK1/MAPK10/MAPK11/MAPK14/MAPK3/MAPK8/  MMP2/MMP9/MTOR/NOTCH1/NOTCH2/NOTCH3/PIK3CA/  PIK3CB/PIK3R1/PRKACA/PTK2/RAF1/SRC/TP53 |
